# The Preparation, Functional Properties, and Application of Food-Derived Antioxidants and Anti-Inflammatory Agents

**DOI:** 10.3390/foods13121839

**Published:** 2024-06-12

**Authors:** Jong-Sang Kim

**Affiliations:** Institute of Agricultural Science and Technology, School of Food Science and Biotechnology, Kyungpook National University, Daegu 41566, Republic of Korea; jongsangkim@gmail.com

This Special Issue of *Foods* titled, “The Preparation, Functional Properties, and Application of Food-Derived Antioxidants and Anti-inflammatory Agents,” has unveiled a fascinating panorama of the multifaceted ways food can contribute to our well-being. We embarked on this journey by exploring the potential of everyday ingredients. The study on sweet potato [1] demonstrated that cooking methods like steaming and deep-frying can significantly enhance its antioxidant capacity. It was previously reported that the physiological function of caffeoylquinic acid derivatives with the plural caffeoyl group, including 3-mono-O-caffeoylquinic acid (chlorogenic acid), 3,4-di-O-caffeoylquinic acid, 3,5-di-O-caffeoylquinic acid, 4,5-di-O-caffeoylquinic acid, and 3,4,5-tri-O-caffeoylquinic acid, is more potent than that of caffeoylquinic acid derivatives with a monocaffeoyl group. This paves the way for the development of novel functional foods readily available in our kitchens [2,3].

Moving beyond individual ingredients, this Special Issue explored the targeted application of specific food components for health benefits. Research on *Euonymus alatus* leaf extract [4] offered promising results in managing Alzheimer’s disease symptoms, while studies on fruit peel polyphenols [5] revealed their potential to combat hyperlipidemia. These findings underscore the prodigious potential of natural food compounds as tools for disease prevention and management.

The exploration then ventured into the gut, a cornerstone of human health. A research study on porcine intestinal mucosal peptides [6] provided valuable insights into their ability to reduce inflammation and improve gut health. The gastrointestinal mucosa maintains a delicate balance between immune tolerance toward dietary components and commensal bacteria. This balance becomes disrupted in Inflammatory Bowel Disease (IBD). Dietary peptides, however, offer a promising avenue for restoring gastrointestinal homeostasis due to their range of biological activities. In vitro studies have extensively evaluated the anti-inflammatory, antioxidant, and immunomodulatory properties of peptides derived from various food sources. Under both healthy and IBD-like inflammatory conditions, animal models have shown these peptides’ protective mechanisms through the modulation of the immune response, specifically influencing the pro-inflammatory/anti-inflammatory and tolerogenic profiles. However, robust clinical trials are needed to translate these promising findings into the human context and definitively establish the role of dietary peptides as functional therapeutic agents for IBD.

This aligns perfectly with the intriguing study on quercetin [7] included in this Special Issue, which suggests it may enhance the effectiveness of cancer treatments by influencing cellular pathways. These findings underscore the intricate link between dietary choices, gut health, and overall disease resistance.

Finally, this Special Issue emphasized the potential of fermented foods, a dietary staple in many cultures. A systematic review [8] highlighted their ability to enhance antioxidant activity, suggesting a possible role in reducing the risk of neurodegenerative diseases. This emphasizes the need for further research into the complex interplay between fermented foods and brain health.

In conclusion, this Special Issue serves as a powerful reminder that food is not merely sustenance; it is a potent tool for promoting health and potentially combating a variety of diseases. From optimizing cooking methods to harnessing the power of specific food components, exploring the wonders of fermentation, and even investigating how dietary choices can influence treatment efficacy, the articles presented here offer a glimpse into the exciting future of food science and its impact on human health. As we move forward, continued exploration in this field holds the promise of unlocking a natural arsenal for promoting health and well-being.
